# Leakage-aware machine learning for data-driven performance prediction of metal–organic framework systems

**DOI:** 10.3389/fmed.2026.1943564

**Published:** 2026-09-28

**Authors:** Nidal H. Abu-Hamdeh, Mohammed N. Ajour, Ahmad H. Milyani, Khalid H. Almitani, Saeed Shirazian

**Affiliations:** 1 Center of Research Excellence in Renewable Energy and Power Systems/Energy Efficiency Group, Department of Mechanical Engineering, Faculty of Engineering, King Abdulaziz University, Jeddah, Saudi Arabia; 2 Department of Electrical and Computer Engineering, Faculty of Engineering, King Abdulaziz University, Jeddah, Saudi Arabia; 3 Department of Mechanical Engineering, Faculty of Engineering, King Abdulaziz University, Jeddah, Saudi Arabia; 4 Institute of Research and Development, Duy Tan University, Da Nang, Vietnam; 5 School of Engineering & Technology, Duy Tan University, Da Nang, Vietnam

**Keywords:** cell viability, drug delivery, drug loading, gradient boosting, metal–organic frameworks, partial least squares

## Abstract

Metal–organic frameworks (MOFs) are highly tunable porous materials whose performance is governed by complex interactions among structural, chemical, material, and operating variables. This study develops a leakage-aware, data-driven framework for predicting two distinct MOF performance endpoints: loading capacity and cell viability. The curated datasets comprised 161 loading-capacity observations described by 110 metal, ligand, functionality, and molecular-fingerprint variables, and 444 cell-viability observations described by 25 MOF, cell-category, and exposure variables. Histogram-based gradient boosting regression (HGBR) and partial least squares regression (PLSR) were optimized using differential evolution within five-fold grouped cross-validation. Exact duplicate records were constrained to the same data partition to prevent information leakage, while an additional source-held-out test was employed to assess transferability to publications not represented during model training. On the duplicate-safe 20% holdout, HGBR achieved the strongest predictive performance for cell viability (*R*^2^ = 0.774, RMSE = 11.655 percentage points, MAE = 8.153, and AARD = 16.69%), whereas PLSR performed best for loading capacity (*R*^2^ = 0.557, RMSE = 0.345 g/g, and MAE = 0.210 g/g). Permutation analysis identified concentration, Zn and Fe indicators, zeta potential, cell category, and particle size as the most influential variables for viability prediction, while PLSR loading predictions were most sensitive to 2,5-dioxidoterephthalate and Mg-related descriptors. However, negative R^2^ values obtained when entire source publications were held out revealed substantial distribution shift and limited cross-study generalization. Overall, the results demonstrate the value of leakage-aware machine learning for rigorous, data-driven performance prediction and screening of MOF systems, while showing that broader standardized datasets and independent-study validation are essential before reliable predictive deployment.

## 1 Introduction

Metal–organic frameworks (MoFs) have been recognized as nanomaterials with crystalline porous structures assembled from metal ions or clusters and organic linkers. Their high internal surface area, adjustable pore architecture, and chemically addressable surfaces have made them important candidates for gas storage, separation, catalysis, drug release, sensing, and biomedical engineering (1–4). In pharmaceutical delivery, these features enable the encapsulation or adsorption of chemically diverse compounds and permit control of host–guest interactions through metal-node selection, linker substitution, and post-synthetic functionalization (3, 5, 6). MOFs may consequently support high payloads, protection of unstable therapeutic molecules, stimulus-responsive release, and combination therapy. These advantages are accompanied by practical constraints involving aqueous stability, particle uniformity, degradation, ion release, and biological compatibility (6, 7).

Two endpoints are especially relevant during early design of drug delivery formulations via porous materials. Drug loading capacity determines the quantity of active compound associated with the carrier (e.g., MoFs) and is influenced by pore accessibility, surface functionality, molecular size, hydrogen bonding, electrostatics, and framework–drug affinity. All these properties can be tuned for MoFs due to their versatile characteristics (8–10).

Another property for drug carriers is cell viability which describes the biological response under a defined dose, exposure duration, particle size, surface charge, framework chemistry, and cellular context. The controlling variables are therefore related but not interchangeable. A carrier with high loading is not necessarily biologically acceptable, and a favorable viability result at one concentration or cell type does not establish general safety. Treating these endpoints as distinct prediction problems avoids imposing a false row-wise correspondence between experiments collected from different publications.

Conventional development relies on synthesis, loading experiments, physicochemical characterization, and *in-vitro* assays. These procedures are indispensable, but comprehensive exploration becomes expensive when metal nodes, linkers, drugs, formulations, doses, and cell systems are varied simultaneously. Computational models such as machine learning (ML) can help prioritize experiments by learning multivariate associations from accumulated records (11–13). Tree ensembles capture nonlinear thresholds and interactions with limited preprocessing, whereas latent-variable methods can stabilize regression when the number of correlated descriptors is large relative to the number of observations (14–17).

Recent studies have analyzed the same MOF drug-delivery endpoints using increasingly complex learning systems. Huwaimel and Alqarni (18) combined multilayer perceptron, random forest, and quantile-regression base learners in stacking configurations; after principal-component analysis and Water Cycle Algorithm optimization, their QR–MLP model attained test *R*^2^ values of 0.99917 for loading capacity and 0.99111 for cell viability. Qin et al. (19) compared Gaussian process regression, sparse Gaussian process regression, and deep Gaussian process regression, using PCA (Principal Component Analysis) and the Cheetah Optimizer; deep Gaussian process regression produced the best mean cross-validation *R*^2^ values, 0.99878 for loading capacity and 0.99911 for cell viability. Alshehri and Pishnamazi (20) assessed gradient boosting trees, XGBoost, and histogram-based gradient boosting with training-set cross-validation for hyperparameter selection; histogram-based boosting yielded test *R*^2^ values of 0.9924 and 0.9987 for loading capacity and viability, respectively. Although these results demonstrate the predictive potential of nonlinear and ensemble models, direct numerical comparison requires caution because the preprocessing, splitting, and validation units differ. The present study therefore emphasizes duplicate-safe grouped validation and an additional publication-held-out stress test rather than relying only on a random row-level split (21–23).

The present study provides a comprehensive modeling workflow based on two models: histogram-based gradient boosting regression (HGBR) and partial least squares regression (PLSR) for prediction of drug loading as well as toxicity of MoF materials in drug delivery applications. Differential evolution (DE) was used for hyperparameter optimization. The novelty is the combination of models and optimizer for prediction of MoF data with possible generalization of the model for future studies in design of advanced formulations for drug delivery.

## 2 Materials and methods

### 2.1 Data source and analysis

The data were reconstructed from the supplementary tables accompanying Wang et al. (11). Their reported data contained drug loading capacity and the cell viability for a wide range of meta organic frameworks. In the dataset, line-wrapped chemical labels were normalized without changing numerical values. It should be pointed out that no observations were removed. Table 1 lists the general overview of dataset and the statistical values. Further details regarding the dataset can be found elsewhere (11).

**Table 1:** Dataset structure and descriptive statistics (11).

Endpoint	Observations	Numeric predictors	Target range	Mean ± SD	Exact duplicates
Cell viability	444	25	2.03–135.14%	82.48 ± 27.20%	13
Drug loading capacity	161	110	0.002–4.200 g/g	0.369 ± 0.456 g/g	1

The viability matrix included five metal indicators (Cu, Zr, Zn, Cr, and Fe), twelve linker or functional-group indicators, four cell-category indicators, zeta potential, particle size, exposure time, and concentration. The loading matrix contained sixteen metal indicators, twenty-eight linker or functionality indicators, and sixty-six molecular fingerprint bits. MOF and drug names were treated as identifiers rather than predictors, thereby avoiding high-cardinality labels that could act as shortcuts. The two endpoints had different observation counts and feature spaces and were modeled independently. Figure 1 shows the strongly skewed loading distribution and the broader viability distribution.

**Figure 1:**
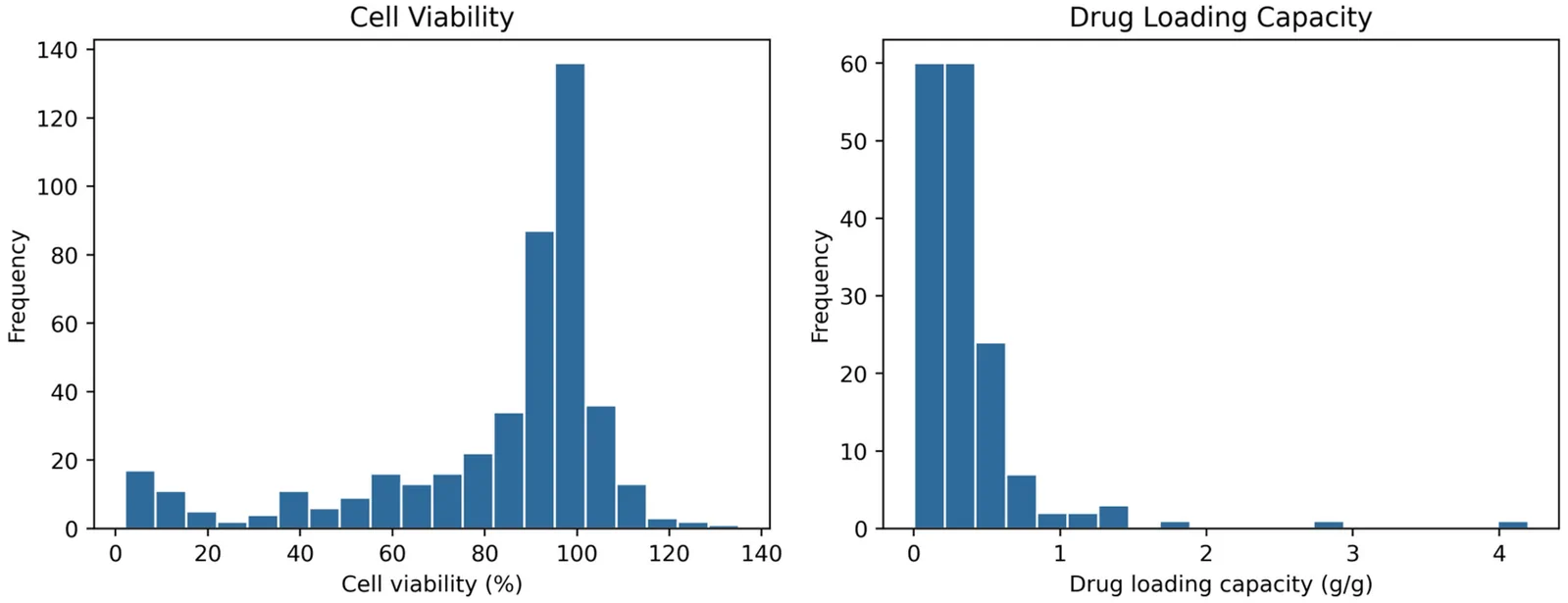
Distributions of the two experimentally reported endpoints (11).

### 2.2 Data quality control and partitioning

No missing numerical values were identified after extraction. Constant predictors were removed within each endpoint before fitting because they contain no estimable information. Exact duplicate records were retained in the archived dataset but assigned a common group identifier. A deterministic grouped 80/20 split was then generated, producing 352 training and 92 test observations for viability and 129 training and 32 test observations for loading capacity. During hyperparameter selection, five-fold GroupKFold cross-validation ensured that duplicate groups remained intact. Preprocessing parameters were fitted within each training fold. The positively skewed loading target was transformed using ln(1 + y) during learning and converted back to g/g for all reported metrics.

A second evaluation was designed as a transfer stress test. Source references/DOIs, rather than duplicate groups, defined the partitions, and 20% of publication groups were held out. Hyperparameters selected in the primary workflow were reused without retuning. This test asks whether relationships learned from one collection of publications generalize to experiments reported by different sources; it is more stringent than ordinary within-distribution validation and was not used to select the final model. Figure 2 shows the general workflow adopted in this study.

**Figure 2:**
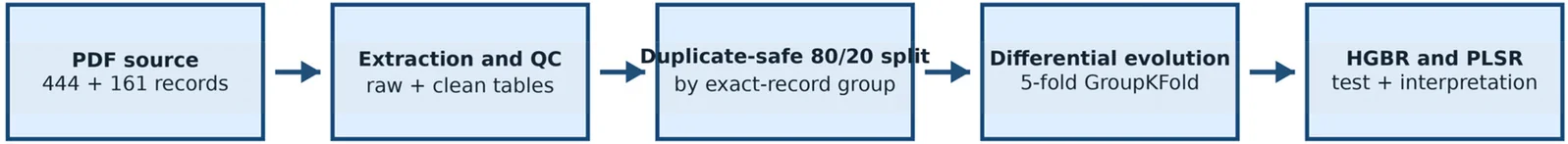
Reproducible extraction, optimization, validation, and interpretation workflow.

### 2.3 Histogram-based gradient boosting regression

HGBR is an additive tree ensemble in which each new regression tree is fitted to reduce the residual loss of the existing ensemble (14, 24). Continuous values are discretized into histogram bins before candidate splits are evaluated, reducing the computational burden relative to checking every observed threshold. For an input vector xᵢ, the prediction after M boosting iterations is written as:

$${\hat{y}}_{i}\;=\;F_{M}(x_{i})\;=\;F_{0}(x_{i})\;+\;\eta \;\overset{M}{\underset{m=1}{\sum }}h_{m}(x_{i})$$ (1)

where F₀ is the initial estimate, hₘ is the mth regression tree, *η* is the learning rate, and M is the number of boosting iterations. At each iteration, the new tree approximates the negative gradient of the squared-error loss. Model capacity is controlled by the maximum number of leaf nodes, minimum observations per leaf, number of iterations, learning rate, and L₂ regularization. These controls are important for small tabular datasets because unrestricted trees can reproduce noise and publication-specific patterns.

Operationally, HGBR first maps continuous predictors into a finite set of ordered bins and accumulates first- and second-order loss information within each bin. Candidate splits are evaluated from these aggregated statistics, so the computational cost depends more strongly on the number of bins than on the number of unique observed values. The sequential trees correct systematic errors left by the current ensemble, while shrinkage controls the contribution of each tree. Maximum leaf count governs interaction complexity; minimum leaf size prevents decisions supported by very few MOF records; and L₂ regularization reduces unstable leaf values. This combination is appropriate for cell viability because dose, particle size, surface charge, metal identity, and cell category can interact through nonlinear thresholds.

### 2.4 Partial least squares regression

PLSR projects correlated predictors into a limited number of supervised latent components before performing regression (15–17). It is suitable for the loading dataset because the descriptor count is high relative to the sample count and many binary fingerprints are correlated. The predictor matrix X and response vector y are decomposed as:

$$X\;=\;TP^{T}\;+\;E,\;\;\;\;y\;=\;Uq^{T}\;+\;f$$ (2)

where T and U are predictor and response score matrices, *P* and q are loadings, and E and f are residual terms. Each component is obtained by selecting weight vectors that maximize the covariance between predictor and response scores:

$${max}_{\|w{\|}_{2}=1}\;\;cov^{2}\left(Xw,\;yc\right)$$ (3)

For loading capacity, PLSR was fitted to ln(1 + y), which reduces leverage from the 4.2 g/g maximum while preserving the ordering and positivity of predictions after inverse transformation.

Unlike unsupervised principal-component regression, PLSR constructs each latent direction using both X and y, favoring combinations of descriptors that explain response-relevant covariance. After extracting a component, the explained predictor and response variation is deflated before the next component is estimated. The final coefficient vector is obtained by projecting the latent-space regression back to the original standardized descriptors. Selecting too few components can underfit chemically meaningful structure–loading relationships, whereas too many components may reproduce noise or rare fingerprint patterns. Grouped cross-validation was therefore used to select component count exclusively from the training partition, and all scaling parameters were re-estimated within each fold to prevent information leakage.

### 2.5 Differential-evolution hyperparameter optimization

Differential evolution (DE) is a population-based global optimizer that does not require gradients and can search continuous and integer-valued hyperparameters (25). For candidate vector xᵢ at generation g, mutation generates:

$$v_{i}^{g}\;={\;x}_{r{}_{1}}^{g}\;+\;F\left(x_{r{}_{2}}^{g}\;-{\;x}_{r{}_{3}}^{g}\right)$$ (4)

where r₁, r₂, and r₃ are distinct population indices and F is the mutation scale. Binomial crossover combines the mutant and target vectors, and selection retains the candidate with the lower objective value. The objective was mean five-fold grouped-CV RMSE divided by the training-target standard deviation. HGBR search variables were learning rate, iterations, maximum leaf nodes, minimum samples per leaf, and L₂ regularization. For PLSR, DE selected the number of latent components. The optimization trace is shown in Figure 3.

**Figure 3:**
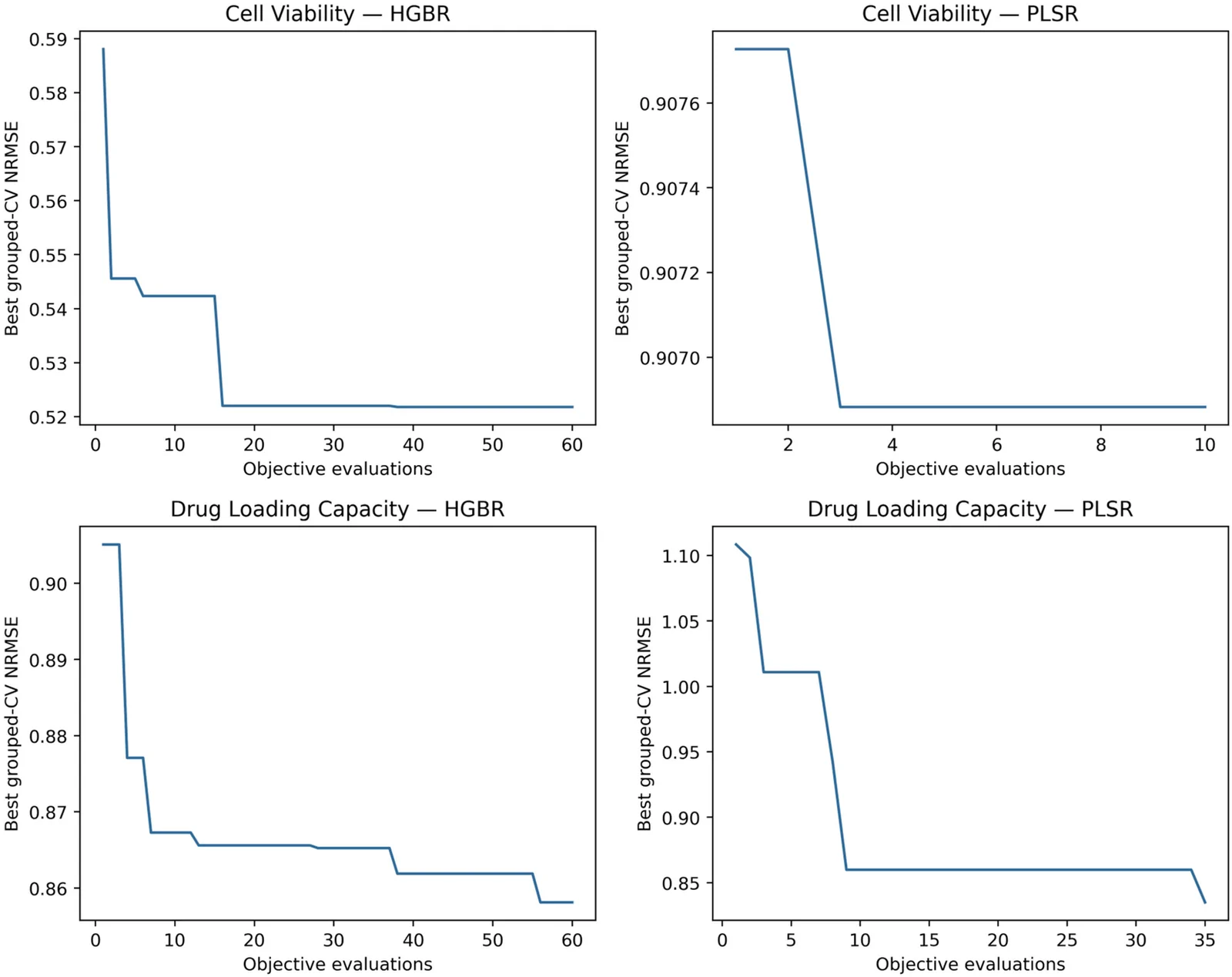
Best grouped-cross-validation objective value during differential-evolution optimization.

Following mutation, binomial crossover selects coordinates from the mutant vector with probability CR and retains coordinates from the target vector otherwise, with at least one mutant coordinate enforced. Each trial configuration is decoded to admissible integer or continuous hyperparameters, evaluated through grouped cross-validation, and replaces its parent only when the normalized RMSE objective improves. The differential weight F controls the magnitude of population differences and therefore the search radius, whereas CR determines how strongly mutant information is mixed into each trial. A fixed seed and bounded search space made the optimization trace reproducible; importantly, the untouched test data and the publication-held-out stress-test data were never used by DE.

### 2.6 Performance measures and interpretation

Predictions were evaluated using the coefficient of determination, root mean squared error, mean absolute error, and average absolute relative deviation:

$$R^{2}\;=\;1\;-\;\frac{\sum_{i=1}^{n}(y_{i}\;-\;{\hat{y}}_{i}{)}^{2}}{\sum_{i=1}^{n}(y_{i}\;-\;\hat{y}{)}^{2}}$$ (5)

$$\mathrm{RMSE}\;=\;\sqrt{\frac{\sum_{i=1}^{n}(y_{i}\;-\;{\hat{y}}_{i}{)}^{2}}{n}},\;\;\;\;\mathrm{MAE}\;=\;\frac{\sum_{i=1}^{n}|y_{i}\;-\;{\hat{y}}_{i}|}{n}$$ (6)

$$\mathrm{AARD}\;(\text{\%})\;=\;\frac{100}{n}\overset{n}{\underset{i=1}{\sum }}\left|\frac{y_{i}\;-\;{\hat{y}}_{i}}{y_{i}}\right|$$ (7)

The model, optimization, and performance formulations are presented in Equations 1–7. Because several loading values approach zero, AARD can become extremely large even when absolute errors are moderate; RMSE, MAE, and *R*^2^ were therefore interpreted jointly. Model sensitivity was evaluated using held-out permutation importance. Each predictor was randomly permuted thirty times, and the mean increase in RMSE was recorded. The analysis measures predictive dependence under the fitted model and should not be interpreted as evidence of causality (26). All computations used Python, NumPy, pandas, SciPy, and scikit-learn (27).

## 3 Results

### 3.1 Optimized configurations

DE algorithm was used for optimization of hyperparameters to improve the accuracy of ML models in this study. Differential evolution selected endpoint-specific model capacities (Table 2). The viability HGBR used smaller leaves and substantially weaker regularization than the loading model, permitting nonlinear dose and material interactions. Conversely, the loading HGBR required strong L₂ shrinkage, consistent with the limited sample size and sparse high-dimensional descriptors. PLSR selected six components for cell viability but only two for loading under grouped cross-validation.

**Table 2:** Hyperparameters selected by differential evolution.

Endpoint	Model	Optimized hyperparameters
Cell viability	HGBR	learning rate 0.09418; 170 iterations; 23 leaf nodes; minimum leaf size 6; L₂ = 0.00436
Cell viability	PLSR	6 latent components
Loading capacity	HGBR	learning rate 0.05164; 110 iterations; 20 leaf nodes; minimum leaf size 20; L₂ = 26.8651
Loading capacity	PLSR	2 latent components; ln(1 + y) target transformation

### 3.2 Primary duplicate-safe performance

The two endpoints favored different model families (Table 3). For cell viability, HGBR achieved a test R^2^ of 0.774, RMSE of 11.655 percentage points, MAE of 8.153, and AARD of 16.69%. PLSR was appreciably weaker (test *R*^2^ = 0.280), demonstrating that viability contains nonlinear thresholds and interactions not captured by a small linear latent space. The training *R*^2^ of HGBR (0.970) exceeded its grouped-CV R^2^ (0.723), showing some optimism in the fitted training score; however, the independent duplicate-safe test score remained close to the grouped-CV estimate.

**Table 3:** Reproducible model performance. Test values are from the duplicate-safe 20% holdout.

Endpoint	Model	Evaluation	*n*	*R* ^2^	RMSE	MAE	AARD (%)
Cell viability	HGBR	Grouped CV	352	0.723	14.636	9.258	32.05
Cell viability	HGBR	Held-out test	92	0.774	11.655	8.153	16.69
Cell viability	PLSR	Grouped CV	352	0.164	25.439	18.135	90.80
Cell viability	PLSR	Held-out test	92	0.280	20.807	15.540	42.99
Loading capacity	HGBR	Grouped CV	129	−0.053	0.448	0.224	228.69
Loading capacity	HGBR	Held-out test	32	−0.044	0.530	0.256	301.32
Loading capacity	PLSR	Grouped CV	129	0.071	0.421	0.222	181.70
Loading capacity	PLSR	Held-out test	32	0.557	0.345	0.210	341.77

For drug loading capacity, PLSR performed better than HGBR. Its test *R*^2^ was 0.557, RMSE was 0.345 g/g, and MAE was 0.210 g/g. HGBR did not improve on the test-set mean (*R*^2^ = −0.044), suggesting that the available 161 observations do not support stable nonlinear partitioning across 110 sparse descriptors. PLSR benefited from supervised dimension reduction, although its grouped-CV *R*^2^ was only 0.071. This difference indicates that the single holdout happened to be more predictable than several cross-validation folds and cautions against interpreting the test result alone. AARD exceeded 300% for both loading models because very small denominators strongly amplify percentage errors. Figure 4 visualizes the held-out observed–predicted agreement and the corresponding residual distributions for both endpoints.

**Figure 4:**
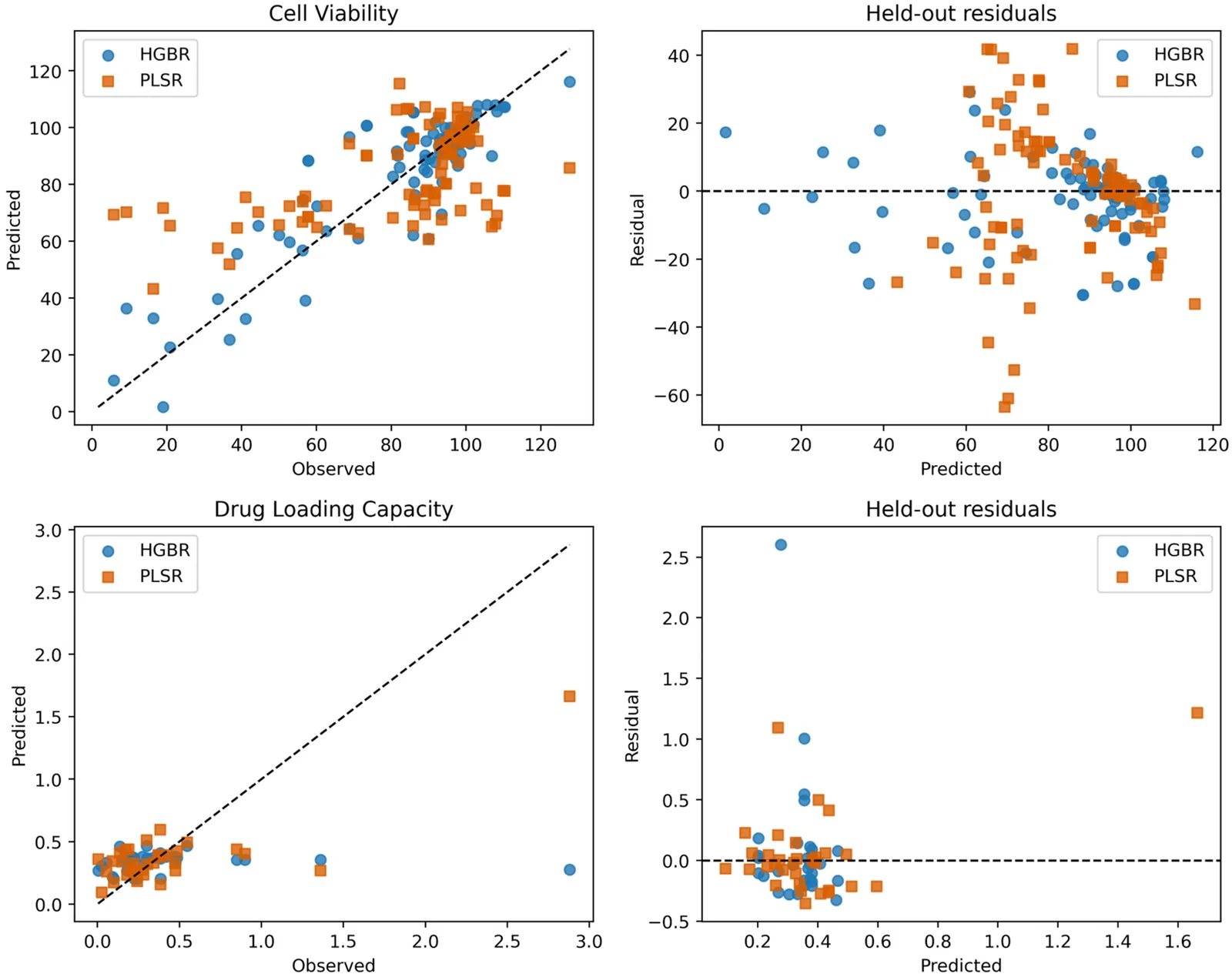
Held-out observed–predicted agreement and residual distributions.

### 3.3 Publication-held-out stress test

Performance deteriorated sharply when complete source publications were excluded from training. Viability R^2^ values were −0.391 for HGBR and −0.119 for PLSR; loading-capacity *R*^2^ values were −0.342 and −0.679, respectively. Negative R^2^ indicates that prediction error exceeded the error obtained by using the mean of the corresponding stress-test target values. This result is not a contradiction of the primary holdout: the tests answer different questions. Duplicate-safe row partitioning estimates interpolation among the material and experimental regimes represented across the compiled data, whereas publication-held-out testing probes transfer under shifts in synthesis, measurement, formulation, and reporting practices. The stress-test outcome therefore limits use of the models to within-domain prioritization and demonstrates that the dataset is not yet adequate for claims of publication-independent external prediction. The complete publication-held-out performance metrics are summarized in Table 4.

**Table 4:** Transfer stress test with entire publication groups held out.

Endpoint	Model	*n*	*R* ^2^	RMSE	MAE	AARD (%)
Cell viability	HGBR	135	−0.391	27.324	19.432	60.60
Cell viability	PLSR	135	−0.119	24.500	19.468	54.20
Loading capacity	HGBR	30	−0.342	0.196	0.158	207.57
Loading capacity	PLSR	30	−0.679	0.220	0.178	218.34

### 3.4 Predictive associations

Permutation importance for the best primary model of each endpoint is presented in Figure 5. In the viability HGBR, concentration caused the largest increase in RMSE when permuted, followed by Zn, zeta potential, Fe, normal-cell status, and particle size. These variables are physically plausible because dose controls cellular exposure, while metal chemistry, surface charge, and size can influence dispersion, uptake, degradation, and ion release. Nevertheless, the rankings may also encode study design: particular MOFs, cell classes, or concentration ranges are clustered within publications. The analysis therefore identifies variables used by the model rather than isolated biological mechanisms.

**Figure 5:**
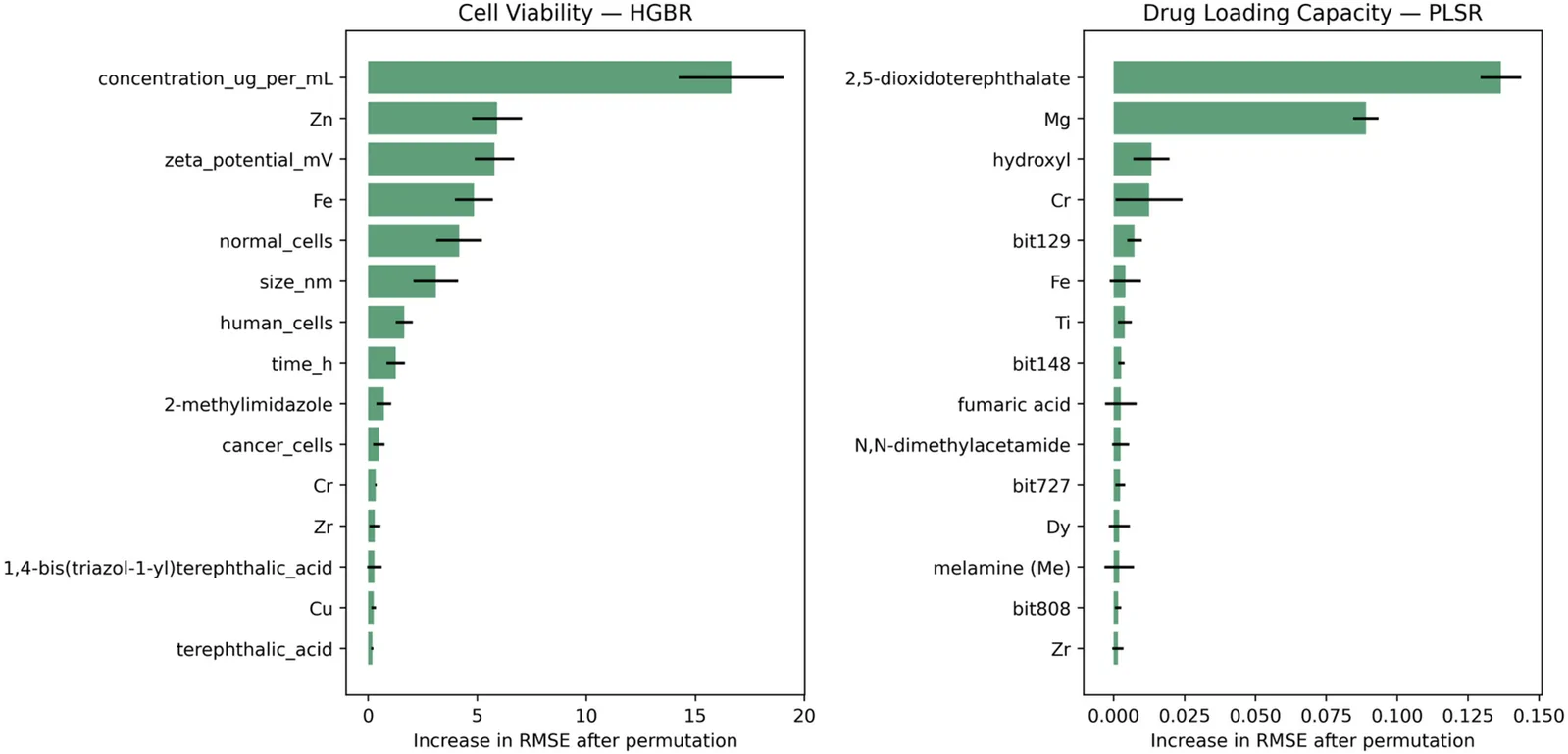
Held-out permutation importance for the best primary model of each endpoint.

For loading-capacity PLSR, 2,5-dioxidoterephthalate and Mg indicators had the largest permutation effects, followed by hydroxyl functionality, Cr, and selected molecular-fingerprint bits. The result is consistent with the importance of framework chemistry and drug–host interactions, but it should be interpreted cautiously because rare binary indicators can acquire high leverage in a small dataset. The weak cross-validation performance and source-transfer failure show that these associations require confirmation using standardized prospective measurements.

## 4 Discussion

The principal finding is that model suitability depends on the endpoint and descriptor regime. Cell viability was better represented by nonlinear histogram-based boosting, which can express concentration thresholds and interactions among composition, surface charge, size, exposure time, and cell category. Loading capacity, in contrast, benefited from PLSR because its 110-column input matrix is high dimensional relative to 161 observations. Supervised latent components can pool correlated fingerprints and chemical indicators, whereas the heavily regularized HGBR was unable to form reliable local partitions.

The primary accuracy is lower than the very high values sometimes reported for literature-derived MOF models. This should not be viewed as a computational defect. Exact duplicates were prevented from crossing partitions, hyperparameters were selected only within training data, and fitted-training performance was separated from cross-validated and held-out results. More importantly, the publication-held-out analysis exposed strong distribution shift. The difference between within-distribution and source-held-out performance illustrates why a single random split can be insufficient evidence for real-world generalization (21–23).

The loading AARD values deserve special attention. AARD divides every absolute error by the corresponding experimental target. Because the dataset includes capacities as low as 0.002 g/g, a modest absolute discrepancy can yield a relative error of hundreds or thousands of percent. RMSE and MAE provide more stable information at this scale. Future studies could supplement these measures with median absolute percentage error, logarithmic error, or target-range-normalized RMSE, while preserving conventional metrics for comparison.

Several limitations arise from the source data. The viability table contains no explicit drug-identity or molecular-fingerprint variable, and the loading table lacks exposure and biological variables; thus the endpoints cannot be combined into a valid two-output model. Literature records also inherit heterogeneity in synthesis, activation, loading protocols, viability assays, cell culture conditions, and reporting. Binary structural indicators do not fully describe pore volume, accessible surface area, framework defects, drug ionization, solvent composition, or release kinetics. Finally, permutation importance is conditional on the fitted model and correlated predictors and cannot establish causation.

A prospective extension should define standardized experimental protocols, record uncertainty and replicate measurements, include quantitative pore and stability descriptors, and reserve entire laboratories or newly generated batches for external validation. Molecular representations could be expanded using graph-based drug and framework embeddings once the sample size supports them. Until such data are available, the present models are best used to rank candidates similar to those already represented, not to replace loading measurements or cytotoxicity testing.

## 5 Conclusion

A fully reproducible modeling workflow was developed for independent prediction of cell viability and drug loading capacity in MOF-based delivery systems. The source supplements yielded 444 cell viability observations with 25 predictors and 161 loading observations with 110 predictors. Differential-evolution optimization selected HGBR as the stronger viability model and PLSR as the stronger loading model. Duplicate-safe test results were *R*^2^ = 0.774 for cell viability and *R*^2^ = 0.557 for loading capacity. Concentration and MOF physicochemical indicators dominated the viability model, whereas selected linker, metal, and molecular descriptors influenced loading prediction. However, all models produced negative *R*^2^ under publication-held-out testing. Accordingly, the framework supports transparent within-domain screening but does not yet justify external predictive claims. The study demonstrates that leakage control, endpoint-specific modeling, and source-level stress testing are essential when machine learning is applied to literature-derived pharmaceutical materials data.

## Data Availability

The original contributions presented in the study are included in the article/Supplementary Material, further inquiries can be directed to the corresponding author.
